# Determination of Free Fatty Acids in Breast Milk Reveals the Presence of Hydroxypalmitic and Stearic Acids

**DOI:** 10.3390/biom14121602

**Published:** 2024-12-14

**Authors:** Maroula G. Kokotou

**Affiliations:** Laboratory of Chemistry, Department of Food Science and Human Nutrition, Agricultural University of Athens, Iera Odos 75, 11855 Athens, Greece; mkokotou@aua.gr; Tel.: +30-2105294261

**Keywords:** breast milk, free fatty acids, HRMS, hydroxy fatty acids, liquid chromatography

## Abstract

Breast milk is a rich source of fatty acids (FAs) while being irreplaceable for the health and development of an infant. Herein, we present a fast and simple method for the direct detection and quantification of 37 free FAs (FFAs) in breast milk samples, avoiding any derivatization step, and a study on the % variation of FA contents in samples collected from the same mother within five consecutive days. The average breakdown of FAs was 60.5% saturated and 39.5% unsaturated, in which polyunsaturated FAs were 13.3% and monounsaturated FAs 26.2%. The most abundant FFA in the breast milk samples was C12:0 (18.3%), followed by C10:0 (15.0%), suggesting that further attention must be paid to the presence and role of medium-chain FAs. Among unsaturated FAs, oleic acid (C18:1 n-9) (13.3%) and linoleic acid (C18:2 n-6) (10.1%) were the most abundant. Remarkable variations of FFA contents within the five consecutive days were observed for C8:0, C10:0, C12:0, C18:1 n-9, and C18:2 n-6. The two isomers α-linolenic acid (C18:3 n-3) and γ-linolenic acid (C18:3 n-6) were quantified in all breast milk samples. The ratio of γ-linolenic acid, which most recently is important for cardiac metabolic maturation, to α-linolenic acid was found to be 1:2. Most importantly, in the present study, we explored the presence of bioactive saturated monohydroxy fatty acids (SHFAs), demonstrating for the first time the existence of distinct hydroxypalmitic and hydroxystearic acids (HPAs and HSAs, respectively) in breast milk.

## 1. Introduction

Breast milk is a precious and invaluable source of food in the first six months of the infant’s life, and sometimes breastfeeding may even prolonged up to two years. Breast milk provides infants not only with energy but also with numerous health benefits, contributing to the development of their immune system and their protection from gastrointestinal, respiratory, and skin diseases, including allergies [1,2]. Among the multitude of nutritive and bioactive components of breast milk, lipids constitute an important class, serving as the main source of energy and as a source of bioactive components [3,4].

The average lipid content in breast milk is approximately 3.8–3.9 g/100 mL, while triglycerides constitute the main fat component [5]. The contents of fatty acids (FAs), including saturated fatty acids (SFAs), monounsaturated (MUFAs), and polyunsaturated (PUFAs), in breast milk do not remain constant. The concentration of FAs present in breast milk depends predominately on the lactation period and the type of milk produced (colostrum, transitional milk, mature milk) and feeding stages (foremilk, hindmilk). In addition, the mother’s diet and the health of the newborn child affect FA composition [5,6,7,8].

The predominant FAs in breast milk are SFAs and MUFAs [5]. Oleic acid (C18:1 n-9) is the most prevalent, constituting around 90% of the MUFAs in breast milk. In contrast, PUFAs constitute only 14–17% of total FAs. Among PUFAs, linoleic acid (C18:2 n-6), belonging to the n-6 family, is the predominant one. α-Linolenic acid (C18:3 n-3), the precursor of the n-3 family of FAs, is present too [5]. Several FAs of breast milk play an important role in the development of the brain and the retina during perinatal development, such as linoleic acid (C18:2 n-6), α-linolenic acid (C18:3 n-3), arachidonic acid (C20:4 n-6), and docosohexaenoic acid (DHA, C22:6 n-3) [5,6].

Mother’s milk is a rich source of FAs for the infant. Although breast milk contains digestive enzymes, including endogenous lipases that may hydrolyze triglycerides, the pancreatic lipase activity is low, and, as a consequence, infants are not able to properly digest long-chain fatty acid triglycerides, releasing FAs [9]. It is known that the absorption of free fatty acids (FFAs) is highly favored in comparison to FAs in the form of triglycerides [10]. For the above reasons, it is important to know the concentrations of FFAs present in breast milk.

However, analytical methods reported in the literature for the determination of FFAs in breast milk are very limited [9,11,12,13]. Gao et al. reported a dried milk spot system for estimating the level of FFAs in human milk collected as dried milk spot [11] and applied it to study the concentration of FFAs in expressed breast milk used in an Australian neonatal unit [12]. Nagasaki et al. developed a Liquid Chromatography/Mass Spectrometry (LC/MS) method for studying lipid profiling in human milk from healthy and mastitic subjects and revealed the presence of unique lipid mediators in human milk [9]. Keilbasa et al. developed a non-derivatization High-Performance Liquid Chromatography/Ultra Violet (HPLC/UV) method for the determination of some n-3 FFAs in breast milk [13].

The aim of this work was the development of a fast and simple analytical method for the detection and quantification of a large set of FFAs in breast milk samples. Herein, we present a Liquid Chromatography-High Resolution Mass Spectrometry (LC-HRMS) method that allows the detection and quantification of FFAs in breast milk samples without the need for tedious and time-consuming sample preparation or derivatization. Furthermore, we discuss the variations of FFAs that were observed in a mother’s milk sample collected in five consecutive days. Finally, in an effort to gain a better insight into the bioactive components of breast milk, we explore for the first time the presence of a class of bioactive oxidized FAs, namely saturated monohydroxy FAs, in human milk.

## 2. Materials and Methods

### 2.1. Chemicals and Reagents

The solvents used were of LC-MS analytical grade and purchased: acetonitrile from Carlo Erba (Val De Reuil, France), isopropyl alcohol, and methanol from Fisher Scientific (Loughborough, UK). Formic acid 98–100% was purchased from Chem-Lab (Zedelgem, Belgium). Caproic acid (C6:0, >98%) was purchased from Alfa Aesar, Lancashire, UK. Heptanoic acid (C7:0, >99.5%), caprylic acid (C8:0, >99.5%), capric acid (C10:0, >99%), myristic acid (C14:0, >99.5%), myristoleic acid (C14:1 n-5, >99%), pentadecanoic acid (C15:0, >99%), margaric acid (C17:0, >98%), linoleic acid (C18:2 n-6, >99%), α-linolenic acid (C18:3 n-3 >99%), γ-linolenic acid (C18:3 n-6, >99%), arachidonic acid (C20:4 n-6, >99), behenic acid (C22:0, >99%) and 4,7,10,13,16,19-*cis*-docosahexaenoic acid (C22:6 n-3, >98%) were purchased from Sigma Aldrich (Steinheim, Germany). Undecanoic acid (11:0, >98%), nonanoic acid (C9:0, >98%), tridecanoic acid (13:0, >98%), 10-*Z*-heptadecenoic acid (C17:1 n-7, >98%), nonadecanoic acid (19:0, >98%), arachidic acid (C20:0, >98%), gadoleic acid (C20:1 n-11, >98%), dihomo-γ-linolenic acid (C20:3 n-6, >98%), 5,8,11-eicosatrienoic acid (C20:3 n-9, >98%), heneicosanoic acid (C21:0, >98%), erusic acid (C22:1 n-9, >98%), 7,10,13,16,19-*cis*-docosapentaenoic acid (C22:5 n-3, >98%), lignoceric acid (C24:0, >98%), tricosanoic acid (23:0, >98%), and nervonic acid (24:1 n-9, >98%) and cerotic acid (26:0, >98%) were purchased from Cayman Chemical Company (Ann Arbor, MI, USA). Lauric acid (C12:0, >99%) was purchased from Acros Organics (Geel, Belgium). Palmitic acid (C16:0, analytical standard), *cis*-9-palmitoleic acid (C16:1 n-7, analytical standard), stearic acid (C18:0, analytical standard), oleic acid (C18:1 n-9, analytical standard), petroselinic acid (C18:1 n-12, analytical standard), and 5,8,11,14,17-*Z*-eicosapentaenoic acid (C20:5 n-3, analytical standard) were purchased from Fluka (Karlsruhe, Germany). The list of analytes is shown in Supplementary Materials (Table S1).

Νine regio-isomers of hydroxypalmitic acid, carrying the hydroxyl group at the positions 2- (2HPA), 3- (3HPA), 6- (6HPA), 7- (7HPA), 8- (8HPA), 9- (9HPA), 10- (10HPA), 11- (11HPA), 16- (16HPA), and nine regio-isomers of hydroxystearic acid, carrying the hydroxyl group at the positions 2- (2HSA), 3- (3HSA), 6- (6HSA), 7- (7HSA), 8- (8HSA), 9- (9HSA), 10- (10HSA), 11- (11HSA), 12-(12HSA) were included in the study. The list of HPAs and HSAs is shown in Supplementary Materials (Table S2).

### 2.2. Stock and Working Solutions

Stock solutions of the standard compounds (1000 mg/L in methanol) were prepared and stored at 4 °C. Working solutions (500 and 1000 ng/mL) were prepared daily by appropriate dilution.

### 2.3. Instrumentation

An ABSciex Triple TOF 4600 (ABSciex, Darmstadt, Germany) was used for the LC-MS/MS studies. The mass spectrometer was connected to a micro-LC Eksigent (Eksigent, Darmstadt, Germany), an autosampler set at (5 °C), and a thermostated column compartment. All the MS experiments were carried out using electrospray ionization (ESI) in negative mode. The data acquisition method process consisted of a TOF-MS full scan (*m*/*z* 50–850) and several information-dependent acquisition (IDA)-TOF-MS/MS product ion scans using 40 V collision energy (CE) with 15 V collision energy spread (CES) used for each candidate ion in each data acquisition cycle (1091). Quantitation (primarily using TOF-MS) and confirmation (using IDA-TOF-MS/MS) were performed in a single run by the workflow followed in the present work. The MS mass spectrometer resolution working conditions were: ion energy 1 (IE1) −2.3, vertical steering (VS1) −0.65, horizontal steering (HST) 1.15, and vertical steering 2 (VS2) 0.00. A Halo C18 microLC HPLC capillary column (particle size 2.7 μm, 90 Å, 0.5 mm × 50 mm) from Eksigent was used. The mobile phase consisted of a gradient (phase A: H_2_O (0.01% formic acid); phase B: acetonitrile (0.01% formic acid)/isopropanol 80/20 *v*/*v*)), and the elution gradient adopted started with 5% of phase B for 0.5 min, gradually increasing to 98% in the next 7.5 min. These conditions were kept constant for 0.5 min, and then the initial conditions (95% solvent B, 5% solvent A) were restored within 0.1 min to re-equilibrate the column for 1.5 min for the next injection (flow rate: 55 µL/min).

### 2.4. Data Processing and Quantification

For the data acquisition, MultiQuant 3.0.2 and PeakView 2.1 from ABSciex (Darmstadt, Germany) were employed. MultiQuant 3.0.2 was also used to record the extracted ion chromatograms (EICs). The base peak chromatograms were constructed for the masses that achieve a 0.01 Da mass accuracy width of 0.01 Da. A margin of ±2.5% was set as the relative tolerance of the retention time.

### 2.5. Method Validation

Breast milk samples were spiked with a mixed standard solution of all analytes to estimate the recovery (%R), matrix factor (MF), and the intra-day variations RSD (%). The recovery was used for the quantification of the analytes in samples.

### 2.6. Sample Preparation

Methanol (4 mL) was added to a screw cap glass centrifuge tube containing breast milk (1 mL). After homogenization by vortex for 30 s, the mixture was centrifuged at 4000× *g* for 10 min. The supernatant (500 µL) was collected and then mixed with water (500 µL) in a vial. This mixture was used for the LC-MS/MS analysis [14].

### 2.7. Breast Milk Samples

3 to 5 mL of breast milk samples were collected by breast pump into tubes and kept at −20 °C until analysis. Before analysis, the frozen samples were left to thaw at room temperature. The samples were collected in the afternoon from the same mother (healthy, 36 years old) 30 days after birth, who gave her informed consent for participation in this study for five consecutive days in order to analyze the variation of FFAs. Mature milk samples originating from the same mother were collected for five consecutive days in order to analyze the variation of FFAs. Breast milk produced about 15 days after birth is specified as mature milk. Mother was fed a normal diet; however, dairy products (milk, cheese, and yogurt) were exclusive of goat origin.

For comparison purposes, commercially available infant formula milk was used. This was specified as organic infant milk from birth (first-age milk for babies). The sample preparation procedure was carried out following the manufacturer’s suggestion.

## 3. Results

### 3.1. Selection of FAs

Our intention in the present work was to study in detail all the saturated FAs, from C6:0 to C24:0, covering not only the usual FAs with even carbon atoms but also including FAs with odd carbon atoms. Usually, FAs with odd carbon atoms are underestimated and have received little attention. However, for some odd carbon atom FAs, for example, C15:0 and C17:0, interesting biological properties have been identified [15,16]. In addition, C26:0 was included in the study. Thus, a large set of medium-chain, long-chain, and very long-chain saturated FAs (twenty in total) are investigated.

Eight FAs containing one double bond were also selected because of their potential bioactivities [17]. In addition to the well-studied oleic acid (C18:1 n-9), we selected myristoleic (C14:1 n-5), palmitoleic (C16:1 n-7), C17:1 n-7, petroselinic (C18:1 n-12), C20:1 n-11, C22:1 n-9, and C24:1 n-9. Finally, we selected the most important usual polyunsaturated FAs (nine in total) containing from 2 to 6 double bonds: linoleic acid (C18:2 n-6), α-linolenic acid (C18:3 n-6), γ-linolenic acid (C18:3 n-3), C20:3 n-6, C20:3 n-9, C20:4 n-6, EPA (C20:5 n-3), C22:5 n-3, and DHA (C22:6 n-3).

**Figure 1:**
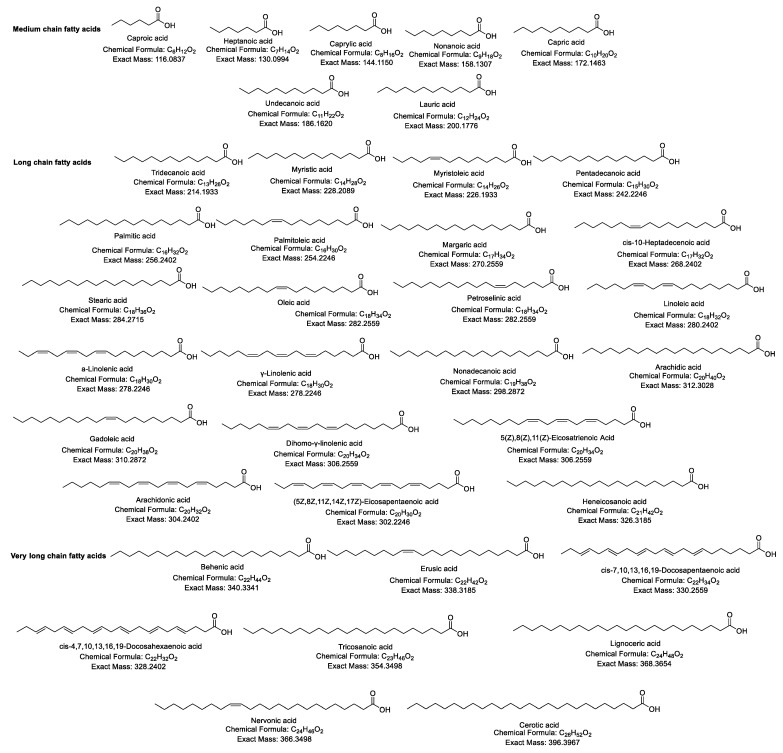
Structures of the standard compounds along with their chemical formulae and exact masses.

The structures of the standard compounds, along with the chemical formulae and exact masses for the common FAs, are summarized in Figure 1.

### 3.2. Method Validation

The breast milk samples were spiked with a mixed standard solution of all analytes (500 ng/mL), and all the experiments were carried out in three replicates. Satisfactory recoveries indicated the accuracy of the proposed method. More specifically, the recoveries ranged from 80 to 112 (Table 1). The precision was investigated by means of the relative standard deviation (%RSD), and the %RSD values ranged from 0.10 to 7.33 (Table 1). The matrix factor was calculated as the ratio of the peak response in the presence of a matrix to the peak response in the pure solvent, and the matrix factor ranged from 0.74 to 1.19 (Table 1). Matrix factor values <1 and >1 denote signal suppression and signal enhancement, respectively.

### 3.3. Chromatography and Determination of FFA Contents

**Figure 2:**
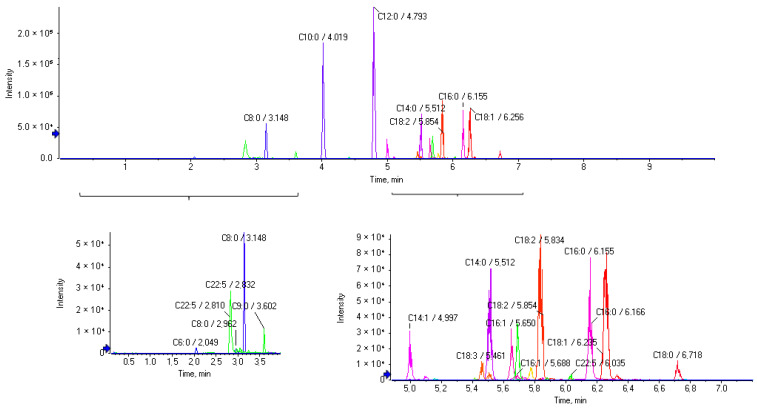
Extracted ion chromatograms (EICs) of a representative breast milk sample. Zoom in on selected areas of the chromatograms is depicted in the lower part of the figure.

Some years ago, we developed an LC-HRMS method for the determination of 22 FFAs in commercial samples of cow and goat milk [14]. We envisioned that such a method could be easily adapted to analyze human breast milk samples, expanding the number of FFAs under investigation. Indeed, a variety of FFAs could be detected and quantified in such samples. Figure 2 shows the extracted ion chromatograms (EICs) of a representative breast milk sample.

**Figure 3:**
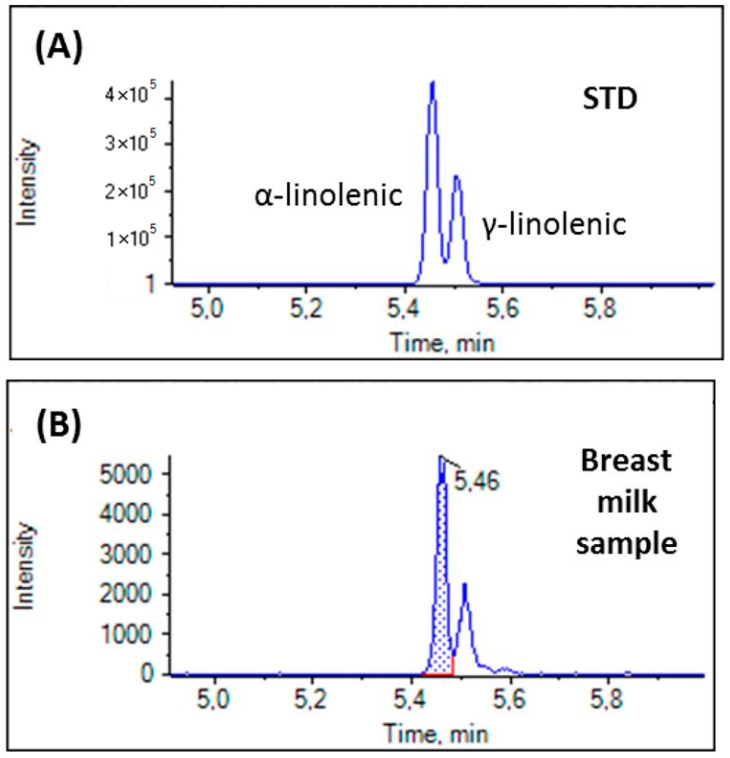
EICs of a mixture of standard solution (1000 ng/mL) (**A**) and a breast milk sample (**B**).

In the present work, we paid special attention to linolenic acid (C18:3), trying to distinguish α-linolenic acid (C18:3 n-3) from γ-linolenic acid (C18:3 n-6), because most recently Paredes et al. have reported that γ-linolenic acid in maternal milk drives cardiac metabolic maturation [18]. The EICs of a mixture of standard solution (A) and a breast milk sample (B) are depicted in Figure 3. The two isomers, α-linolenic acid (C18:3 n-3) and γ-linolenic acid (C18:3 n-6) are separated under the present chromatographic conditions, allowing the quantification of each one of them. EICs of separate standard solutions of α-linolenic acid and γ-linolenic acid are shown in Figure S1 (Supplementary Materials).

The contents of FFAs in breast milk samples, expressed as μg/mL, are summarized in Table 2. Minimum, maximum, and mean values of each FA, together with % composition, for the five days are reported. The most abundant FFA in the breast milk samples studied was found to be the medium-chain C12:0 (18.3%), followed by C10:0 (15.0%). In addition, medium-chain C8:0 was present in a considerable quantity (11.0%).

Oleic acid (C18:1 n-9) was the most prevalent of MUFAs (13.3%), while notable amounts of C14:1 n-5 (3.1%) and C16:1 n-7 (7.5%) were estimated. From the class of PUFAs, linoleic acid (C18:2 n-6) was found to be the most abundant (10.1%). Among the other PUFAs, α-linolenic acid (C18:3 n-3) and γ-linolenic acid (C18:3 n-6) were determined at 1.4% and 0.7% quantities, respectively, while C20:3 n-6 and C20:5 n-3 at 0.6% and 0.4%, respectively.

Collectively, the average breakdown of FFAs was 60.5% SFAs and 39.5% UFAs, of which MUFAs were 26.2% and PUFAs were 13.3%.

### 3.4. Heat Μap and Variations

**Figure 4:**
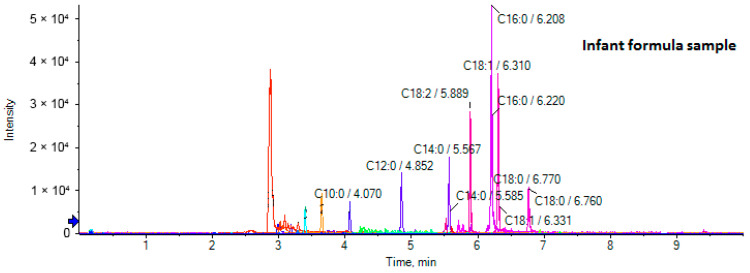
EICs of an infant formula sample.

An infant formula sample was used for comparison purposes. Its EICs are depicted in Figure 4.

**Figure 5:**
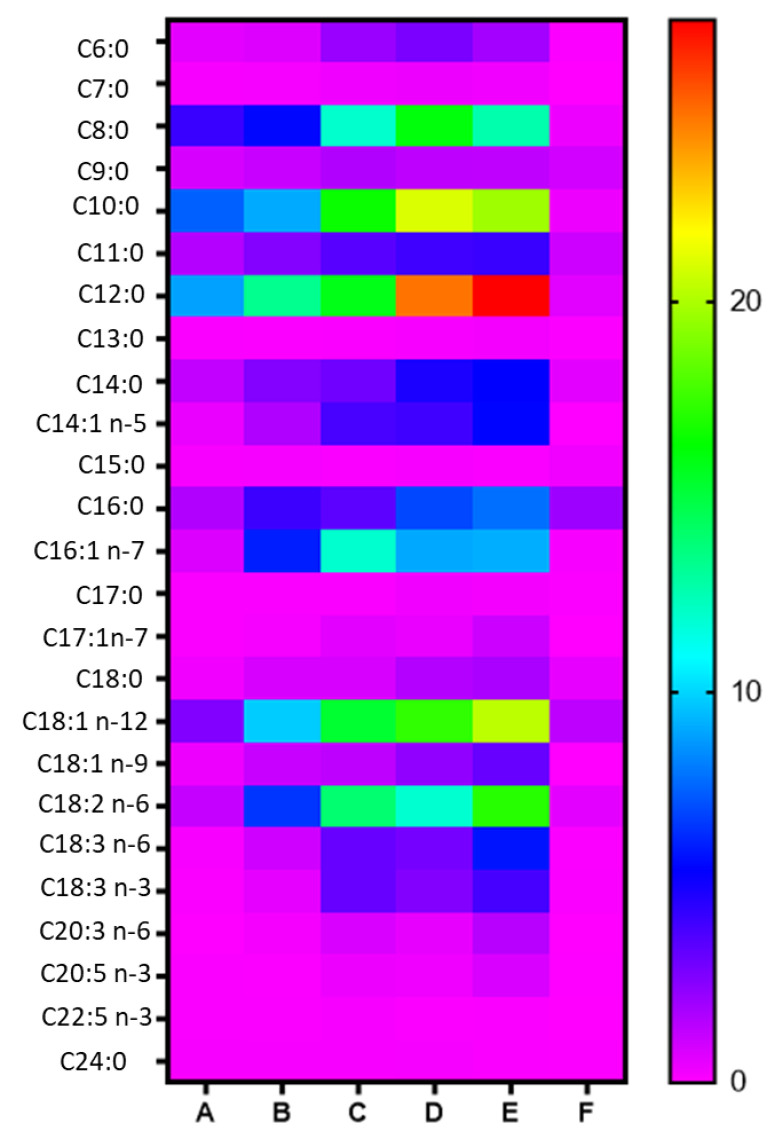
Heat map variations of FFAs in breast milk collected in five consecutive days (A–E), compared to the content of FFAs in an infant formula sample (F). Red and orange tones indicate higher data values, while green and blue tones represent lower data values. Purple tone indicates the lowest data values.

Figure 5 depicts a heat map of the 25 FFAs, which were quantified (μg/mL) in the mother’s breast milk, collected in five consecutive days (A–E), compared to FFAs determined in an infant formula sample (F). Variations were observed for the concentrations of C6:0, C8:0, C10:0, C11:0, C12:0, C14:0, C14:1 n-5, C16:0, C16:1 n-7, C18:1 n-9, C18:1 n-12, C18:2 n-6, C18:3 n-3, and C18: n-6 in the breast milk samples. The most remarkable variation was recorded for the most abundant C12:0, whose quantity was considerably increased within five days. Remarkable variations within the 5 days were also observed for C10:0, C18:1 n-9, and C18:2 n-6. Compared to the infant formula, medium-chain FAs (MCFAs) C8:0, C10:0, C11:0, C12:0, and long-chain FAs C14:0, C14:1 n-5, C16:0, C16:1 n-7, C18:1 n-9, C18:2 n-6, C18:3 n-3, and C18:3 n-6 were found at considerably higher concentrations in breast milk samples, highlighting the higher quality of breast milk. The total amount of FFAs quantified in the breast milk samples ranged from 33 to 152 μg/mL, while in infant formula, the total amount of FFAs was estimated to be 10 μg/mL.

**Figure 6:**
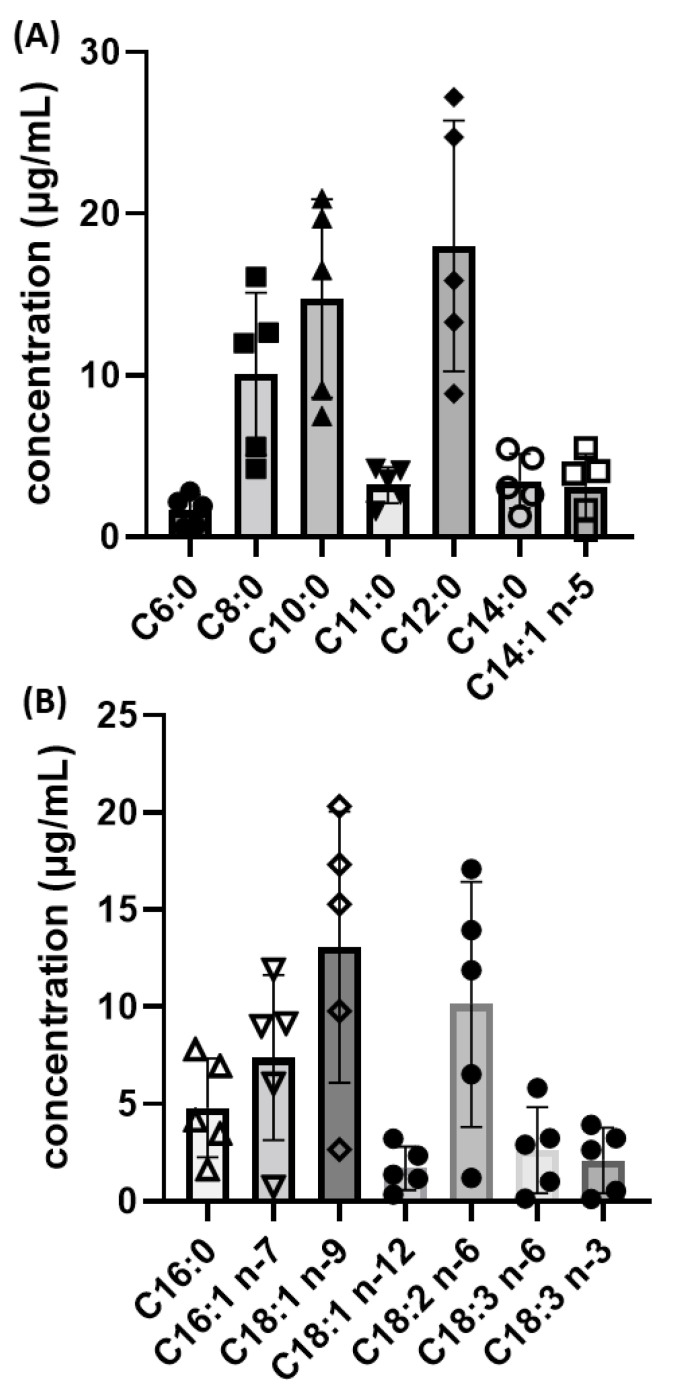
Variations of the amount of (**A**) C6:0, C8:0, C10:0, C11:0, C12:0, C14:0, C14:1 n-5, and (**B**) C16:0, C16:1 n-7, 18:1 n-9, 18:1 n-12, C18:2 n-6, C18:3 n-6, and C18:3 n-3 in breast milk samples within five consecutive days. Graphs were created using GraphPad Prism 9.2.0.

Notable variations within the five consecutive days were observed for fourteen FAs, namely C6:0, C8:0, C10:0, C11:0, C12:0, C14:0, C14:1 n-5, C16:0, C16:1 n-7, 18:1 n-9, 18:1 n-12, C18:2 n-6, C18:3 n-6, and C18:3 n-3. These variations in FFA contents of breast milk samples are better shown in Figure 6.

### 3.5. Exploring the Presence of Saturated Monohydroxy Fatty Acids in Breast Milk

**Figure 7:**
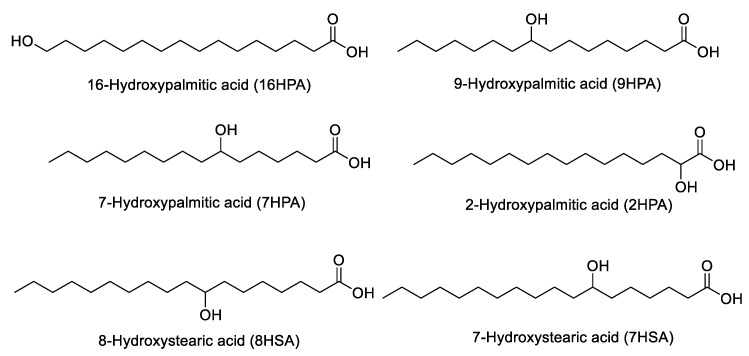
Representative structures of HPAs and HSAs.

We have previously shown that a number of uncommon hydroxylated saturated FAs are present in cow and goat milk, as well as in yogurt [19,20], and that hydroxystearic acids (HSAs) and hydroxypalmitic acids (HPAs) exhibit various bioactivities. Representative structures of HPAs and HSAs are shown in Figure 7. 7HPA and 9HPA inhibit cancer cell proliferation, while 7HSA and 9HSA protect β-cells from cytokine-induced apoptosis [21]. Thus, we decided to explore the presence of such bioactive FAs in breast milk as well as in infant formula.

**Figure 8:**
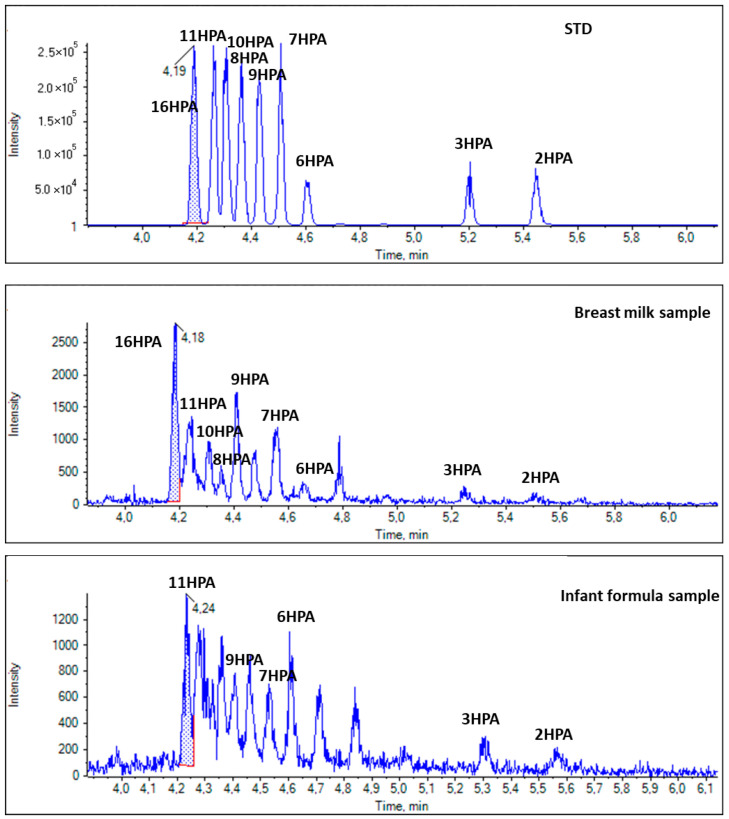
EICs of HPAs (*m*/*z* 271.2279) in a standard solution and representative samples of breast milk and infant formula.

For the first time, we demonstrate that both HPAs and HSAs are present in breast milk. As shown in Figure 8, several HPA regio-isomers were detected in the breast milk sample, such as 16HPA, 11HPA, 10HPA, 9HPA, 8HPA, 7HPA, 6HPA, 3HPA, and 2HPA. In the case of HPAs (*m*/*z* 271.2279), the most intense peak was observed at 4.18 min in the breast milk samples, and this peak suggests the presence of 16HPA based on the retention time of the standard solution sample. For comparison purposes, an infant formula sample was also analyzed. As shown in Figure 8, several peaks corresponding to HPAs (*m*/*z* 271.2279) were observed, but with lower intensities, indicating lower concentrations.

**Figure 9:**
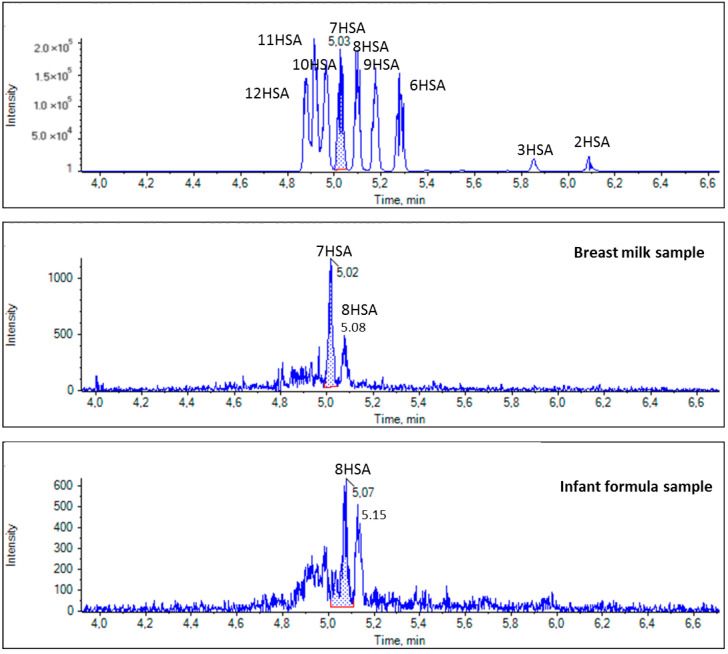
EICs of HSAs (*m/z* 299.2592) in a standard solution and representative samples of breast milk and infant formula.

In the case of HSAs (*m*/*z* 299.2592), 7HSA seemed to be the predominant one in all breast milk samples. Representative EICs shown in Figure 9 demonstrate the presence of 7HSA in a breast milk sample; however, its absence in an infant formula sample.

## 4. Discussion

The determination of the contents of FFAs in breast milk has received little attention up to now. However, FFAs of breast milk require in-depth research because FFAs may be easily absorbed by infants’ organisms, in contrast to FAs incorporated into triglycerides, which require first enzymatic hydrolysis in order to be absorbed in the digestion track. In industrially developed countries, triglycerides constitute the major part (reaching 95%) of dietary fats consumed by humans, and each individual consumes daily around 100 to 150 g of fat. It is known that humans and other animals require the action of pancreatic and gastric lipases as essential enzymes for the efficient digestion of fats [22,23]. For the absorption of fat by the enterocytes, the enzymatic hydrolysis of triglycerides is necessary to release FFAs and monoglycerides. Studies on healthy volunteers without any pancreatic or gastric deficiency have demonstrated the critical physiological contributions of both pancreatic and gastric lipases for the hydrolysis of dietary triglycerides [24]. It has been shown that 66% of triglyceride hydrolysis of a liquid test meal was sufficient for complete absorption, while pancreatic and gastric lipases contributed to 48% and 18%, respectively, hydrolysis of triglyceride acyl chains. However, as mentioned in the introduction, pancreatic and gastric lipase activities are low in infants, and they are not able to properly digest long-chain fatty acid triglycerides [9].

Two lipases are naturally present in human milk, namely bile salt-stimulated lipase (BSSL) and lipoprotein lipase (LPL) [25,26,27,28]. In humans, BSSL is expressed in the mammary glands during lactation and is secreted into the milk. In the presence of bile salts, it has high lipase and esterase activity, being able to hydrolyze triglycerides, diglycerides, monoglycerides, cholesterol esters, phospholipids, galactolipids, and ceramides. LPL is expressed in mammary glands, and it hydrolyzes triglycerides in lipoproteins to release free fatty acids and monoglycerides. BSSL may have an important role in infant fat digestion, while LPL seems not to have a known function in milk [29]. As summarized in a recent review article, the total fat content and total FA composition of expressed breast milk do not seem to be influenced by the storage and handling process [30]. However, triglyceride concentration seems to be reduced by a concomitant increase in FFA levels after being exposed to various conditions, which may be attributed to the action of endogenous lipases. Nevertheless, preservation of the breast milk lipid classes may be achieved for at least up to 5 months after prolonged storage in a deep freezer but not in a domestic freezer [30]. It has to be noticed that Holder pasteurization totally destroys BSSL, and this abolishment reduces the overall fat absorption from human milk by 17–30% [31,32].

Nagasaki et al. studied the contents of 10 long-chain FFAs in breast milk [9] using LC/MS, while Gao et al. studied 11 long-chain FFAs using a dried milk spot system [11]. Kielbasa’s study focused on only three PUFAs (eicosapentaenoic acid (EPA), DHA, and α-linolenic acid) using HPLC/UV [13]. In our work, we have studied 37 FFAs, and we have detected and quantified 25 FFAs in breast milk samples. In addition to usual long-chain FAs, we studied medium-chain FAs (C6:0, C8:0, C10:0, and C12:0) and FAs with odd carbon atoms (C7:0, C9:0, C11:0, C13:0, C15:0, C17:0, and C19:0). Furthermore, several long-chain MUFAs (C14:1 n-5, C16:1 n-7, C17:1 n-7, and C18:1 n-12) were studied.

We observed that C12:0 was the most abundant (18.3%) FFA and, together with C10:0, C8:0, and C6:0, constituted a major part of FFAs, almost half of the total FFAs (45.9%). This finding suggests that we have to pay attention to and further explore the role of MCFAs in breast milk, which is quite underexplored so far. In literature, it has been reported that C10:0, C12:0, and C14:0 are important FAs for the physical needs of a child because they stabilize the bacterial flora in the child’s digestive tract [33,34]. Moreover, they also play a role in the conversion of EPA into DHA [35]. Human milk FAs may be derived from three sources: (a) de novo biosynthesis in the mammary gland, (b) intake of dietary lipids, and (c) mobilization of endogenous adipose or hepatic lipids. As a matter of fact, the human mammary gland may strongly induce de novo lipogenesis upon physiological stimulation. However, such de novo lipogenesis results mainly in the generation of MCFAs because of the presence of a specific thioesterase II enzyme [36,37,38]. Thus, our findings that MCFAs constitute almost half of the total FAs in the breast milk samples studied may be explained by the fact that the de novo lipogenesis in the mammary gland leads mainly to MCFAs. In addition, the high levels of MCFAs in our samples may be attributed in part to the mother’s diet. As we stated in Section 2.7, the mother was fed dairy products of goat origin. We have noticed that goat milk is rich in MCFAs [14,39].

Although oleic acid (C18:1 n-9) was the most prevalent of MUFAs (13.3%), notable amounts of C14:1 n-5 (3.1%) and C16:1 n-7 (7.5%) were estimated in breast milk. Linoleic acid (C18:2 n-6) was found at a slightly lower concentration (10.1%) than oleic acid, while α-linolenic acid (C18:3 n-3) and γ-linolenic acid (C18:3 n-6) were determined at significantly lower concentrations (1.4% and 0.7%).

Accumulated knowledge has led to the conclusion that each particular FA exerts different biological properties, including the regulation of lipolysis and lipogenesis and endocrine signaling, which depends on the length of the aliphatic chain and the saturation or the presence of one or more double bonds [40,41]. FFAs may interact with various free fatty acid receptors, which include FFA1, FFA2, FFA3, and FFA4 (also known as GPR40, GPR41, GPR43, and GPR120, respectively) receptors [40,41].

Although wide knowledge is available for long-chain saturated or unsaturated FAs, limited data on the biological importance of medium-chain FAs are available in the literature. A recent review article summarizes the role and the effect of MCFAs on human health and disease [42]. The antibacterial properties of FAs and the potential of FAs to become new antibacterial agents are of particular interest [43]. Lauric acid (C12:0) stands out among medium-chain and long-chain FAs, and a most recent review article discusses the antimicrobial properties of lauric acid [44]. The antimicrobial property of lauric acid against *Propionibacterium acnes* has rendered it a potential agent to treat inflammatory acne vulgaris [45]. Abel-Anzaku et al. have shown the antibacterial activity of lauric acid on some selected clinical isolates [46], while Yang et al. have demonstrated the inhibition of *Clostridium difficile* growth in vitro and the reduction of inflammation in a mouse infection model [47]. Thus, the presence of lauric acid in breast milk and its high abundance may confer antibacterial properties to breast milk. In addition, other MCFAs, including C6:0, have been reported to promote TH1 and TH17 differentiation, thus playing a role in inflammation [48].

Another relatively underexplored subclass of FAs is the class of monounsaturated FAs. A huge interest has been paid so far to oleic acid; however, limited data are available about the role and biological properties of myristoleic (C14:1 n-5), palmitoleic (C16:1 n-7), C17:1 n-7, petroselinic (C18:1 n-12), C20:1 n-11, C22:1 n-9, and C24:1 n-9. Our findings that C14:1 n-5 and C16:1 n-7 are present in breast milk (3.1% and 7.5%, respectively) are of special interest because both of them exhibit bioactivities. For example, palmitoleic acid has been proposed to prevent or control metabolic and inflammatory disorders [49] and may reduce the severity of invasive group A streptococcal infection [50]. Thus, palmitoleic acid, which was estimated in the present study to be a highly abundant MUFA in breast milk, following oleic acid, may contribute to controlling metabolism and inflammation in infants.

As we mentioned in the introduction, we carefully distinguished α-linolenic (C18:3 n-3) and γ-linolenic acid (C18:3 n-6). Both of them are important bioactive components. α-linolenic acid and its corresponding oxylipins play an important role in human cardiovascular diseases [51], while γ-linolenic acid exhibits anti-inflammatory properties [52]. In particular in infants, γ-linolenic acid in maternal milk plays a critical role in driving cardiac metabolic maturation [18]. In addition, an association of breast milk γ-linolenic acid with infant anthropometric outcomes in urban, low-income Bangladeshi families has been demonstrated [53]. In our study, we showed that γ-linolenic acid was present in breast milk in approximately half quantity in comparison to α-linolenic acid.

In general, the findings of our study regarding long-chain FFAs are in accordance with those of Nagasaki et al. [9], who showed the following long-chain FFA profile in human milk: oleic acid (C18:1 n-9) > linoleic acid (C18:2 n-6) > stearic acid (C18:0) > C16:0 > α-linolenic acid (C18:3 n-3). However, in our study, the concentration of stearic acid was found to be lower than that of palmitic acid. In addition, Nagasaki et al. estimated 5.40 µM C20:4 n-6, 2.94 µM C20:5 n-3, and 3.34 µM C22:6 n-3 [9]. In our samples, we have quantified only C20:5 n-3 (0.4 µg/mL or 1.21 µM) and neither C20:4 n-6 nor C22:6 n-3. As we discussed in the introduction, we have to keep in mind that the concentration of FAs present in breast milk depends not only on the lactation period but also on the mother’s diet and the health of the newborn child [5,6,7,8]. In addition to the studies of Nagasaki et al. [9], Gao et al. [11], and Kielbasa et al. [13], Chuang et al. have estimated the contents of five additional FFAs in breast milk from Taiwanese mothers using a GC/MS method [54]. Thus, to our knowledge, the quantification of free MCFAs C6:0, C8:0, odd-chain FAs C7:0, C9:0, C11:0, C13:0, C15:0, C17:0, and MUFA C14:1 in breast milk is reported in the present study for the first time.

In our previous studies, the existence of uncommon monohydroxylated saturated FAs in dairy products has been explored [19,20]. Following both suspect and targeted lipidomics approaches, we have identified in cow and goat milk, as well as in yogurt, previously unrecognized families of SHFAs [19,20]. Investigating the bioactivities of hydroxystearic acids and hydroxypalmitic acids, we have identified interesting bioactivities for these oxidized FAs, including antiproliferative activity on cancer cells and protection of β-cells from cytokine-induced apoptosis [20]. In the present study, for the first time, distinct HPAs and HSAs are detected in breast milk samples using reference compounds. Although such bioactive FAs are present in minor quantities, they may play a role in contributing to the protection and promotion of the infant’s health.

Collectively, the accumulation of experimental data shows that the lipid composition of breast milk depends on various parameters, including maternal diet, physical condition, delivery mode, neonatal health, and lactation stage. Due to the intricate complexity of the lipid composition of human milk, our understanding of the human milk lipidome remains incomplete, and further research on this fascinating topic is urgently needed because breast milk lipidome may critically influence the health and development of the infant.

## 5. Conclusions

In conclusion, we developed an LC-HRMS method for the determination of FFAs in breast milk samples, which is fast and simple, not requiring a derivatization step. From the 37 FFAs studied by the present method, 25 FFAs were detected and quantified in the samples studied. The most abundant FFA was found to be C12:0 (18.3%), followed by C10:0 (15.0%), suggesting that further research has to be performed in order to understand the role of saturated medium-chain FAs. Oleic acid (C18:1) (13.3%) and linoleic acid (C18:2) (10.1%) were the most abundant UFAs. Remarkable variations of FFA contents were observed for some of the FFAs (C8:0, C10:0, C12:0, C18:1 n-9, and C18:2 n-6) within the five consecutive days examined. Furthermore, an HRMS-based targeted lipidomics approach exploring bioactive oxidized FAs was conducted for the first time in breast milk samples and revealed the presence of several HPAs, as well as 7HSA. Although in minor quantities, such bioactive FAs may contribute to the promotion of an infant’s health, highlighting the importance of breastfeeding. Further studies using a large number of breast milk samples are needed in order to confirm the findings of the present research and to understand the role of each FA in the health and development of an infant.

## Figures and Tables

**Table 1:** Accuracy (recovery %), precision data (RSD %), and matrix factor (MF) in the spiked breast milk samples.

Fatty Acid	Spike Level 500 ng/mL		
	Recovery (%R)	RSD (%)	MF
C6:0	95	1.62	1.10
C7:0	88	3.82	1.13
C8:0	93	0.10	1.19
C9:0	102	0.85	1.14
C10:0	88	2.10	1.24
C11:0	98	2.15	1.11
C12:0	100	5.40	1.37
C13:0	106	2.62	1.19
C14:0	80	2.87	1.16
C14:1 n-5	110	0,60	1.03
C15:0	108	0.98	0.74
C16:0	109	3.07	1.11
C16:1 n-7	101	4.28	1.16
C17:0	104	2.80	0.82
C17:1 n-7	106	1.00	0.82
C18:0	100	3.99	1.13
C18:1 n-9	108	2.62	0.89
C18:1 n-12	95	1.52	0.90
C18:2 n-6	101	2.10	1.10
C18:3 n-6	104	1.40	1.10
C18:3 n-3	93	1.23	1.04
C19:0	96	0.90	0.77
C20:0	96	1.98	0.94
C20:1 n-11	112	6.87	0.38
C20:3 n-6	107	1.51	0.60
C20:3 n-9	89	2.03	0.87
C20:4 n-6	108	3.10	1.20
C20:5 n-3	107	1.58	1.11
C21:0	90	2.54	0.75
C22:0	90	6.08	1.31
C22:1 n-9	87	4.21	0.61
C22:5 n-3	112	6.10	0.88
C22:6 n-3	111	3.81	0.92
C23:0	90	4.27	0.84
C24:0	88	1.20	1.14
C24:1 n-9	97	1.50	0.64
C26:0	98	7.33	0.77

**Table 2:** Contents of FFAs in breast milk samples (μg/mL milk) (triplicates).

Free Fatty Acid	Minimum Value (μg/mL)	Maximum Value (μg/mL)	Mean Value ± SD (μg/mL)	%
C6:0	0.6	2.8	1.6 ± 0.5	1.6
C7:0	0.1	0.3	0.2 ± 0.1	0.2
C8:0	4.2	16.1	10.8 ± 0.2	11.0
C9:0	0.9	1.6	1.3 ± 0.3	1.3
C10:0	7.5	20.9	14.8 ± 0.8	15.0
C11:0	1.6	4.2	3.2 ± 0.3	3.3
C12:0	8.9	27.2	18.0 ± 4.0	18.3
C13:0	0.1	0.3	0.2 ± 0.1	0.2
C14:0	1.3	5.4	3.2 ± 0.1	3.2
C14:1 n-5	0.4	5.5	3.1 ± 1.1	3.1
C15:0	0.1	0.2	0.1 ± 0.0	0.1
C16:0	1.6	7.8	4.8±1.2	4.9
C16:1 n-7	0.7	11.9	7.4 ± 1.1	7.5
C17:0	0.1	0.3	0.2 ± 0.1	0.2
C17:1 n-7	0.1	1.1	0.6 ± 0.1	0.6
C18:0	0.3	1.8	1.1 ± 0.4	1.1
C18:1 n-9	2.7	20.3	13.1 ± 6.1	13.3
C18:1 n-12	0.3	3.2	1.7 ± 1.1	1.7
C18:2 n-6	1.2	17.1	10.0 ± 2.4	10.1
C18:3 n-6	0.1	2.5	1.4 ± 0.3	1.4
C18:3 n-3	0.1	1.2	0.7 ± 0.2	0.7
C19:0	<LOQ	<LOQ	-	0
C20:0	n.d.	n.d.	-	0
C20:1 n-11	n.d.	n.d.	-	0
C20:3 n-6	0.1	1.5	0.6 ± 0.2	0.6
C20:3 n-9	n.d.	n.d.	-	0
C20:4 n-6	<LOQ	<LOQ	-	0
C20:5 n-3	0.1	0.8	0.4 ± 0.1	0.4
C21:0	n.d.	n.d.	-	0
C22:0	n.d.	n.d.	-	0
C22:1 n-9	n.d.	n.d.	-	0
C22:5 n-3	0.1	0.2	0.1 ± 0.1	0.1
C22:6 n-3	n.d.	n.d.	-	0
C23:0	n.d.	n.d.	-	0
C24:0	0.1	0.2	0.1 ± 0.1	0.1
C24:1 n-9	n.d.	n.d.	-	0
C26:0	n.d.	n.d.	-	0

n.d.; not detected, <LOQ; lower of limit of quantification, %; percentages of total free fatty acids.

## Data Availability

All data are available in the main text or the Supplementary Materials.

## Supplementary Materials

The following supporting information can be downloaded at: https://www.mdpi.com/article/10.3390/biom14121602/s1, Table S1: List of common fatty acids together with their exact masses [M-H]^−^, their retention time R_t_ (min), and their limits of detection (LOD) and quantification (LOQ). Table S2: List of HFAs together with their exact masses [M-H]^−^, their retention time R_t_ (min), and their limits of detection (LOD) and quantification (LOQ). Figure S1. EICs of standard solutions of α-linolenic acid and γ-linolenic acid (500 ng/mL).
