# Supplementary material for: Determination of Free Fatty Acids in Breast Milk Reveals the Presence of Hydroxypalmitic and Stearic Acids
Source: Biomolecules. 2024 Dec 14;14(12):1602. doi: 10.3390/biom14121602 (PMC11674463; doi:10.3390/biom14121602)
Supplement: Supplementary file 1 [file biomolecules-14-01602-s001.zip › biomolecules-3278017-supplementary.pdf]

Supplementary Material

Determination of Free Fatty Acids in Breast Milk Reveals the Presence of Hydroxypalmitic and Stearic Acids

Maroula G. Kokotou

Laboratory of Chemistry, Department of Food Science and Human Nutrition, Agricultural University of Athens, Iera Odos 75, 11855 Athens, Greece; mkokotou@aua.gr;

Tel.: +30-2105294261

Table of contents

1. Table S1. List of common fatty acids together with their exact masses $[M-H]^-$, their retention time R_t (min), and their limits of detection (LOD) and quantification (LOQ).

2. Table S2. List of HFAs together with their exact masses [M-H]-, their retention time Rt (min), and their limits of detection (LOD) and quantification (LOQ).

3. Figure S1. EICs of standard solutions of α-linolenic acid and γ-linolenic acid (500 ng/mL).

Table S1. List of analytes together with their exact masses $[M-H]^-$, their retention time R_t (min), and their limits of detection (LOD) and quantification (LOQ).

Analyte	$[M-H]^-$	R_t (min)	LOD (ng/mL)	LOQ (ng/mL)
Caproic acid (C6:0) [1]	115.0765	2.1	0.5	1.5
Heptanoic acid (C7:0)	129.0921	2.7	0.4	1.3
Caprylic acid (C8:0) [1]	143.1078	3.2	0.5	1.1
Nonanoic acid (C9:0)[2]	157.1234	3.7	0.5	1.2
Capric acid (C10:0) [1]	171.1391	4.1	0.5	1.7
Undecanoic acid (11:0)	185.1547	4.5	0.6	1.7
Lauric acid (C12:0) [1]	199.1704	4.9	0.6	1.8
Tridecanoic acid (13:0)	213.1860	5.3	0.5	1.5
Myristic acid (C14:0) [1]	227.2017	5.6	0.6	1.8
Myristoleic acid (C14:1 n-5) [1]	225.1850	5.1	0.6	1.8
Pentadecanoic acid (C15:0) [1]	241.2173	6.0	0.8	2.4
Palmitic acid (C16:0) [1]	255.2330	6.3	0.9	2.3
cis-9-Palmitoleic acid (C16:1 n-7) [1]	253.2173	5.8	1.6	4.8
Margaric acid (C17:0) [1]	269.2486	6.6	0.8	2.4
cis-10-Heptadecenoic acid (C17:1 n-7) [1]	267.2330	6.2	0.8	2.4
Stearic acid (C18:0) [1]	283.2643	6.8	0.9	2.8
Oleic acid (C18:1 n-9) [1]	281.2486	6.4	0.7	2.3
Petroselinic acid (18:1 n-12)	281.2486	6.3	0.6	2.1
Linoleic acid (C18:2 n-6) [1]	279.2330	6.0	0.6	1.8
a-Linolenic acid (C18:3 n-6)	277.2173	5.46	0.6	1.8
γ-Linolenic acid (C18:3 n-3)	277.2173	5.52	0.6	1.7
Nonadecanoic acid (19:0)	297.2799	7.1	0.5	1.5
Arachidic acid (C20:0) [1]	311.2956	7.0	0.8	2.4
Gadoleic acid (C20:1 n-11)	309.2799	6.9	0.7	1.2
Dihomo-γ-linolenic acid (C20:3 n-6) [1]	305.2486	6.1	0.6	1.8
5,8,11-Eicosatrienoic acid (C20:3 n-9)	305.2486	6.2	0.6	1.8
Arachidonic acid (C20:4 n-6) [1]	303.2330	5.9	0.6	1.8
cis-5,8,11,14,17-Eicosapentaenoic acid (C20:5 n-3) [1]	301.2173	5.6	0.6	1.8
Heneicosanoic acid (C21:0)	325.3112	7.5	0.5	1.9
Behenic acid (C22:0)[2]	339.3269	7.7	0.8	2.4
Erusic acid (C22:1 n-9)	337.3112	7.3	0.6	1.8
cis-7,10,13,16,19-Docosapentaenoic acid (C22:5 n-3) [1]	329.2486	6.1	0.4	1.2
cis-4,7,10,13,16,19-Docosahexaenoic acid (C22:6 n-3) [1]	327.2330	5.9	0.4	1.2
Tricosanoic acid (23:0)	353.3425	7.9	0.4	1.2
Lignoceric acid (C24:0)	367.3582	8.0	0.5	1.4
Nervonic acid (24:1 n-9)	365.3425	7.7	0.6	1.3
Cerotic acid (26:0)	395.3895	8.3	0.5	1.5

Table S2. List of HFAs together with their exact masses $[M-H]^-$, their retention time R_t (min), and their limits of detection (LOD) and quantification (LOQ).

Analyte	$[M-H]^-$	R_t (min)	LOD (ng/mL)	LOQ (ng/mL)
16HPA[3]	271.2279	4.19	0.9	2.6
11HPA[3]	271.2279	4.25	0.5	1.6
10HPA[3]	271.2279	4.31	0.5	1.2
9HPA[3]	271.2279	4.43	0.6	1.7
8HPA[3]	271.2279	4.36	0.5	1.2
7HPA[3]	271.2279	4.51	0.8	2.4
6HPA[3]	271.2279	4.60	0.7	1.3
3HPA[3]	271.2279	5.20	0.5	1.4
2HPA[3]	271.2279	5.45	0.3	0.8
12HSA[3]	299.2592	4.88	0.5	1.4
11HSA[3]	299.2592	4.92	0.6	1.3
10HSA[3]	299.2592	4.97	0.4	1.1
7HSA[3]	299.2592	5.03	0.4	1.1
8HSA[3]	299.2592	5.09	0.5	1.0
9HSA[3]	299.2592	5.18	0.6	1.8
6HSA[3]	299.2592	5.28	0.7	2.1
3HSA[3]	299.2592	5.85	0.3	0.9
2HSA[3]	299.2592	6.09	0.1	0.4

References

1. Kokotou, M. G., Mantzourani, C., Kokotos, G. (2020). Development of a liquid chromatography-high resolution mass spectrometry method for the determination of free fatty acids in milk. *Molecules*, *25*, 1548.
2. Mantzourani, C., Batsika, C. S, Kokotou, M. G., Kokotos, G. (2022). Free fatty acid profiling of Greek yogurt by liquid chromatography-high resolution mass spectrometry (LC-HRMS) analysis. *Food Research International*, *160*, 111751.
3. Kokotou, M. G., Mantzourani, C., Bourboula, A., Mountanea, O. G., Kokotos, G. (2020). A liquid chromatography-high resolution mass spectrometry (LC-HRMS) method for the determination of free hydroxy fatty acids in cow and goat milk. Molecules 25(17), 3947.

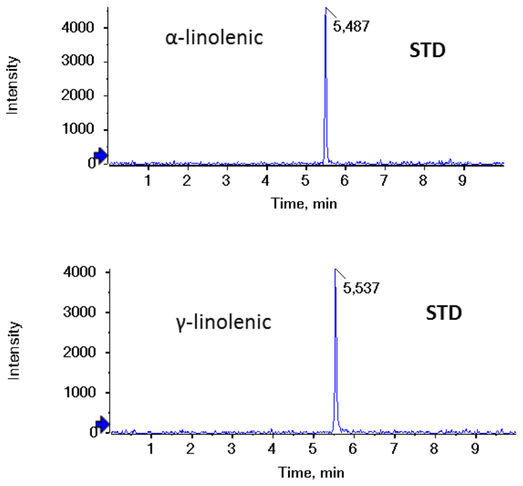

Figure S1. EICs of standard solutions of α-linolenic acid and γ-linolenic acid (500 ng/mL).
